# Functional, not Taxonomic, Composition of Soil Fungi Reestablishes to Pre-mining Initial State After 52 Years of Recultivation

**DOI:** 10.1007/s00248-022-02058-w

**Published:** 2022-07-12

**Authors:** Julien Roy, Rüdiger Reichel, Nicolas Brüggemann, Matthias C. Rillig

**Affiliations:** 1 Institut Für Biologie, Ökologie Der Pflanzen, Freie Universität Berlin, 14195 Berlin, Germany; 2 Brandenburg Institute of Advanced Biodiversity Research, 14195 Berlin, Germany; 3 Institute of Bio- and Geosciences, Forschungszentrum Jülich GmbH, Agrosphere (IBG-3), 52425 Jülich, Germany

**Keywords:** Fungal functional guilds, Nutrient stoichiometry, Soil biodiversity loss, Soil reclamation, Succession, Temporal dynamic

## Abstract

Open-cast mining leads to the loss of naturally developed soils and their ecosystem functions and services. Soil restoration after mining aims to restore the agricultural productivity in which the functions of the fungal community play a crucial role. Whether fungi reach a comparable functional state as in the soil before mining within half a century of recultivation is still unanswered. Here, we characterised the soil fungal community using ITS amplicon Illumina sequencing across a 52-year chronosequence of agricultural recultivation after open-cast mining in northern Europe. Both taxonomic and functional community composition showed profound shifts over time, which could be attributed to the changes in nutrient status, especially phosphorus availability. However, taxonomic composition did not reach the pre-mining state, whereas functional composition did. Importantly, we identified a positive development of arbuscular mycorrhizal root fungal symbionts after the initial three years of alfalfa cultivation, followed by a decline after conversion to conventional farming, with arbuscular mycorrhizal fungi being replaced by soil saprobes. We conclude that appropriate agricultural management can steer the fungal community to its functional pre-mining state despite stochasticity in the reestablishment of soil fungal communities. Nonetheless, conventional agricultural management results in the loss of plant symbionts, favouring non-symbiotic fungi.

**Supplementary Information:**

The online version contains supplementary material available at 10.1007/s00248-022-02058-w.

## Introduction

Open-cast mining leads to the loss of soil and the important ecosystem functions and services soil provides [1], especially for agriculture. Reclamation strategies are key to reestablishing healthy soils [2]. While reclamation strategies are employed after these disturbances, it is poorly studied whether fungal communities respond to soil management and reach pre-mining initial state. Soil fungi are a major component of the soil community supporting soil functions and plant productivity, which is key to maintain productive agriculture and food security [3–5]. For instance, fungi with saprotrophic ability participate in the decomposition of soil organic matter and thus control the recycling, release or retention of nutrients essential to plant nutrition. Fungi with mycorrhizal ability take up nutrients in soil and directly transfer these nutrients to plants through root associations. Fungi can also cause disease (pathotrophy) and directly decrease plant productivity. Finally, some fungi can also colonise roots without a mycorrhizal structure or disease symptoms and can perform symbiotic, parasitic and saprotrophic activity.

Improving the knowledge on the temporal trajectory of the soil fungal community following disturbance can deliver important insights into how soil functions driven by these organisms shift over time and how they can be steered to a desired state with local-scale management, such as plant recultivation and nutrient addition. How the soil fungal community changes over time at scales from decades to centuries to millennia can be addressed by studying chronosequences [6]. Chronosequences are landscape-level sequences of soils for which a process has started at different points in time. Ideally, those different soils are spatially close to ensure a common species pool, on the same bedrock and in the same climate. Chronosequences provide important insights into how soil fungal diversity is linked to soil nutrient status, nutrient availability and soil development or degradation over time, which in turn offers opportunities to manipulate successional processes for restoration purposes [7].

During conventional agricultural land use, important changes in soil properties, such as nutrient availability, nutrient retention or soil aggregation, occur [8]. Fertilisation is a widespread practice to overcome limitation of plant productivity by nutrient availability. Nitrogen (N) and phosphorus (P) fertilisation affects fungal functional guilds differently [9]. For instance, the abundance, diversity and composition of arbuscular mycorrhizal (AM) fungi, a key group of root fungal symbionts involved in plant nutrition and resistance to abiotic and biotic stresses [10], depend on soil P or N limitation and their stoichiometric ratio [11–14] resulting in a mutualistic or parasitic outcome of the symbiosis [15]. Moreover, conventional tillage and pesticide application are conducted to reduce weed and the occurrence of fungal pathogens. These practices impose strong ecological and evolutionary selection on arbuscular mycorrhizal (AM) fungi [16] and fungal pathogens in agroecosystems (e.g. the selection for resistance, [17] with serious threats to crop yield and human health [5]. While tillage, fertilisers and pesticides have been repeatedly shown to strongly harm the AM fungal community [18–21], how multiple fungal functional guilds respond to such treatments and potentially replace each other is poorly known.

The comparison of the end-point of a chronosequence with the pre-disturbance state allows to test whether soil management can steer the ecosystem to a desirable state, or whether it is possible reach the pre-disturbance state, and to evaluate the degree of stochasticity in the reassembly of communities. Some studies have shown divergent community reassembly following disturbance in comparison with the pre-disturbance state [6]. On the other hand, the functional composition of these communities followed predictive reassembly [6] consistent with niche theory [22]. More studies in various ecological contexts need to be conducted to address predictability of the reassembly of communities and for soil fungal communities in particular.

Here, we analysed the taxonomic and functional guild composition of the soil fungal community using ITS amplicon Illumina sequencing across 52 years of agricultural recultivation after open-cast lignite mining in central Europe. We specifically addressed (1) how the soil fungal community changes over time in relation to soil nutrient status, nutrient availability and soil development or degradation over time and (2) the more general potential of soil management to steer the ecosystem to a desirable state and to reach the pre-disturbance state. Building on previous results at the same sites, we raised two hypotheses. First, we predicted that the taxonomic composition of the soil fungal community fails to reach the pre-mining state, as evidenced for soil bacteria [23], but that the fungal functional guild composition will, as predicted by niche theory [22]. Second, we hypothesised the replacement of AM fungi by soil saprotrophs [9, 12].

## Methods

### Chronosequence Description

**Fig. 1:**
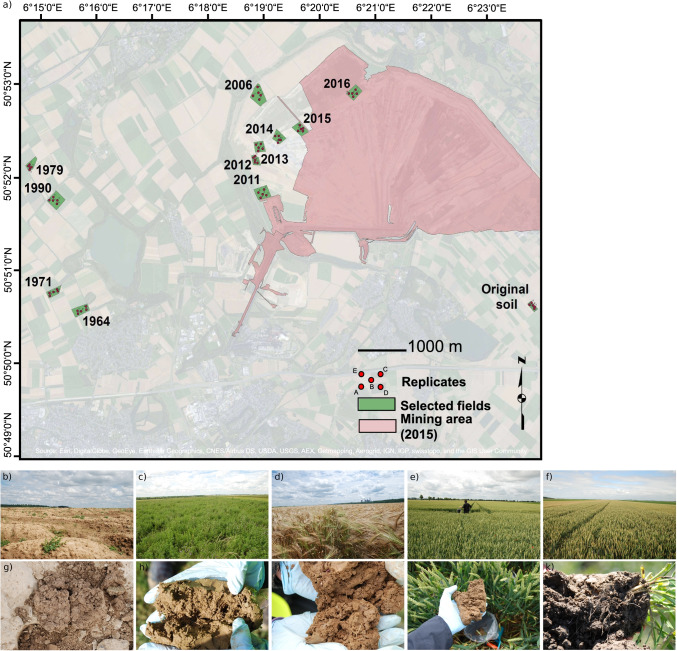
Spatial distribution of the selected fields across the recultivation chronosequence and images of the fields and of their respective soils across the different phases of the recultivation process. (a) Map of the selected fields across the chronosequence. The year since when the substrate has been deposited and the recultivation process started is indicated. The red dots indicate the sampling points in each field. Fields cultivated since 2016 (b, g; beginning of phase 1), 2013 (c, h; end of phase 1), 2011 (d, i; phase 2), 1964 (e, j; end of phase 3) and original soil before mining (f, k)

The study sites correspond to agricultural fields located within an area of 25 km^2^ (6°15′0’ E to 6°21′0’ E and 50°50′5’ N to 50°53′0’ N) (Fig. 1a) of an open-cast lignite mine between Cologne, Aachen, Mönchengladbach and Düsseldorf [12, 23, 24]. Mean annual temperature and rainfall are 9.8 °C and 829 mm, respectively. The mean elevation above sea level is 124 ± 22 m. Soil is a loess loam. Lignite is extracted after removing the former field soil and subjacent bedrock layers by RWE Power AG (Essen, Germany) up to 200 m deep. During extraction, field soil and loess parent material are mixed and deposited at the backside of the mine. This substrate is used as the basis for agricultural recultivation (Fig. 1b, g). Recultivation starts after three months of settling of the dumped substrate and levelling by bulldozers. During the first three years, fields are permanently covered by alfalfa (Fig. 1c, h; freshly deposited substrate and alfalfa fields are hereafter referred to as “phase 1”). These fields never receive biocide treatments during this period but an initial fertilisation (30 kg per hectare of N, 30 kg per hectare of P_2_O_5_ and 30 kg per hectare of K_2_O). At the end of phase 1, green waste compost is applied at a rate of 40 tons per hectare. Then, alfalfa permanent culture is converted to conventional agriculture. Conventional agricultural management is resumed with wheat or barley cropping for two years (hereafter referred to as “phase 2”, winter barley at sampling time, Fig. 1d, i). In addition to 20 kg N from the previous alfalfa culture, an average amount of 41 kg N, 143 kg P_2_O_5_ and 273 kg K_2_O get available for plant production over four years from the applied compost. In addition, there is an average mineral fertilisation of 167 kg N, 150 kg P_2_O_5_ and 120 kg K_2_O per hectare in order to raise the soil nutrients to the level of the soil before mining. Finally, fields are returned to farmers and conventionally managed following area typical agricultural practices and plant protection guidelines in accordance with the German Fertilizer Ordinance (hereafter referred to as “phase 3”, Fig. 1e, j). The selected fields were ploughed to a soil depth of 30 cm and planted with winter wheat in the year of sampling. The previous crop rotation was dominated by winter barley, sugar beet or winter rapeseed. In the winter wheat seasons, the fields received on average a mineral fertilisation of 198 kg N, 80 kg P_2_O_5_ and 60 kg K_2_O per hectare. This three-phase recultivation process in the lignite mining area has not changed fundamentally over the last few decades, providing a chronosequence of fields recultivated for less than one year to 52 years, allowing to study the temporal dynamic of the soil fungal community under the conditions of this lignite mine. Other agricultural fields that have not been subject to extraction (hereafter referred to as “pre-mining phase”, Fig. 1f, k) reflect the original soil and fungal community state before mining to be compared to fields subject to the recultivation process.

### Sample Collection

We surveyed 12 fields, one per year since recultivation, with increasing time since recultivation spanning the three recultivation phases and the pre-mining phase (Fig. 1). The sampling was performed in two different seasons, in winter (March 2016) and summer (June 2016). Sampling occurred within two days. Phase 1 fields correspond to fields restored in 2016, 2015, 2014 and 2013 (freshly deposited substrate in 2016 was available only in the summer season) at time of sampling. Phase 2 fields correspond to fields restored in 2012 and 2011 at time of sampling. Phase 3 fields correspond to fields restored in 2006, 1990, 1979, 1971 and 1964 at time of sampling. Pre-mining phase corresponds to one field that had not been subject to extraction. The average field size was ∼6 ha. We sampled five soil replicates per field, keeping a minimum distance of 25 m from the borders: four samples from each field corner and one from its centre, with an average distance of 107 ± 26 m in between [12]. Each replicate contained five pooled soil cores of 10 cm depth and 6 cm diameter, which originated from a square of 5 m × 5 m around the sampling GPS point. The resulting 115 soil samples were sieved to 2 mm and stored at –80 °C until DNA extraction. Nine soil physicochemical variables and potential emissions of two greenhouse gases were measured for each sample (details in [12]).

### ITS2 Illumina Sequencing

Soil fungal community profiling follows established protocols [25]. Briefly, soil DNA was extracted from 0.25 g using the PowerSoil DNA isolation kit (MoBio Laboratories Inc., Carlsbad, CA, USA), following the manufacturer’s instructions. The fungal ITS2 genomic region was amplified by PCR using the fITS7 (5′‐GTGARTCATCGAATCTTTG‐3′) and the ITS4 primers (5′‐TCCTCCGCTTATTGATATGC‐3′) [26]. The purified equimolar-pooled amplicon library was sequenced using 300‐bp paired‐end Illumina MiSeq 2000 sequencing (Illumina Inc., San Diego, CA, USA) at the Berlin Centre for Genomics in Biodiversity Research (BeGenDiv, Berlin, Germany).

### Bioinformatics Analyses

Denoised, chimaera-free, non-singleton amplicon exact sequence variants (ESVs) were obtained using the dada2 R package [27]. Taxonomic annotation of ESVs was performed using IDTAXA [28] in DECIPHER [29] against UNITE [30], keeping only fungal ESVs. Functional guilds of ESVs were retrieved using the FUNGuild database [31]. Raw reads are available at ENA under study project PRJEB51095. Fungal ESV sequences are available at ENA under accession numbers OV986018-OV989728. The dataset, including the ESV contingency table, the sample metadata and the taxonomic and functional annotation of ESVs, is available at figshare (10.6084/m9.figshare.20160578).

Prior to statistical analysis of the fungal community composition, sequencing depth among samples was rarefied to 33,000 reads per sample. Four samples were removed due to too low sequencing depth, keeping a final total of 111 samples. To analyse the functional community composition, the guild annotation matrix was transformed to a ‘trait’ matrix in which, for each ESV, the membership of an ESV to a particular guild was coded present (1) or absent (0). Based on this trait matrix, we calculated the proportion of ESVs in each community that belong to a particular functional guild, that is, the community functional potential. The proportion of ESVs for each functional guild was weighted by the relative abundance of those ESVs in the community. Note that because a taxon can be attributed to more than one functional guild, the sum of the proportion of ESVs of each functional guild within a community does not sum up to 1.

### Statistical Analyses

All statistical analyses were conducted in R [32]. The variation in soil physicochemical variables was analysed using principal component analysis using dudi.pca() in ade4 [33]. Community composition analyses were performed using vegan [34]. To analyse the variation in ESV and guild community composition, dissimilarities in ESV community composition and community functional potential were calculated using Bray–Curtis dissimilarity using vegdist(). Dissimilarities were visualised using NMDS ordination with metaMDS() and soil physicochemical variables were fitted to the scores of the communities in the NMDS space using envfit(). The proportion of variance in community dissimilarities attributed to recultivation phase, year of recultivation, season, was analysed using permutational multivariate ANOVA [35] using adonis2(). In addition, the relative importance (marginal R^2^) of each soil physicochemical variable was calculated using permutational multivariate ANOVA. Finally, we performed an indicator species analysis to statistically support the selection of different ESVs across years, using multipatt() in indicspecies [36]. P-values were adjusted for multiple testing using the false discovery rate [37].

## Results

**Fig. 2:**
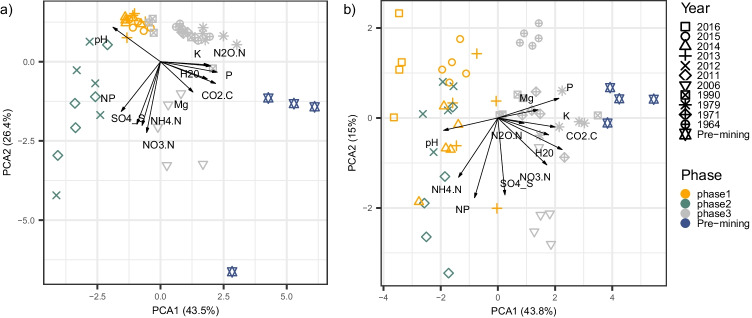
Agricultural fields from different phases of the recultivation chronosequence have major differences in soil physicochemical properties. The figure depicts a biplot of a principal component analysis (PCA) of soil physicochemical variables, (a) in winter, (b) in summer. Symbols represent the year when recultivation started after mining and soil deposition. Colours represent the main management phases of the recultivation process. Direction and length of the arrows represent the direction of increasing value and the contribution of the variable to the principal components, respectively. The percentage of explained variance for each axis is displayed

We first performed a principal component analysis to visualise the changes in soil physicochemical parameters across recultivation phases and years within phases. In both winter and summer, soil physicochemical characteristics strongly differed between each phase of the recultivation (Fig. 2). Soil pH was more alkaline at the beginning of the recultivation (phase 1) while all other parameters were low. Ammonium (NH_4_^+^) and sulphate (SO_4_^2−^) soil content was higher after conversion to the barley cropping (phase 2). Soil water content (likely due to an increased water-holding capacity as result of organic matter enrichment), potassium (K) and total soil phosphorus (P) content were higher at the end of the chronosequence, after decades of conventional agriculture by local farmers (phase 3), and even more in the pre-mining field. The main differences between winter and summer was the NO_3_^−^ soil content, higher in winter in phase 2, but higher in summer in phase 3 and pre-mining fields, probably reflecting the difference in the period of fertilisation between the fields managed by RWE company (phase 2) and the fields managed by local farmers (phase 3 and pre-mining fields). Soil greenhouse gas emission potential did not differ between seasons: N_2_O and CO_2_ potential emissions were higher in phase 3, and even more in the pre-mining field, compared to phases 1 and 2.

**Fig. 3:**
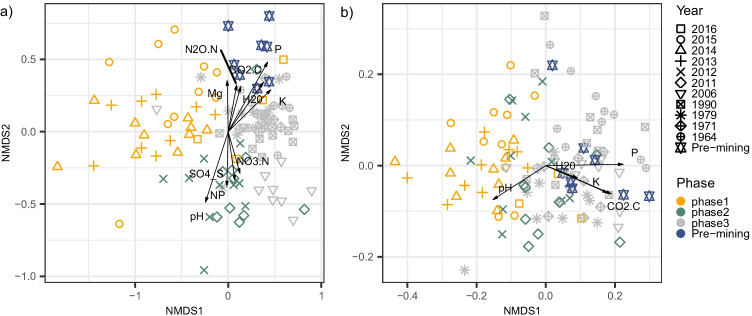
The taxonomic and guild composition of the soil fungal community varies among agricultural fields of different phases of the recultivation chronosequence and correlates with soil physicochemical properties. The figure depicts an NMDS ordination of Bray–Curtis dissimilarities of (a) ESV and (b) functional guild community composition of soil fungi across the recultivation chronosequence. Symbols represent the year when recultivation started after mining and soil deposition. Colours represent the main management phases of the recultivation process. The correlation with soil physicochemical variables is overlaid onto the ordination. Direction and length of the arrows indicate direction of increasing value of the variable and strength of the correlation with sample scores in the ordination, respectively. Significant correlations (*p* < 0.001) are displayed

**Fig. 4:**
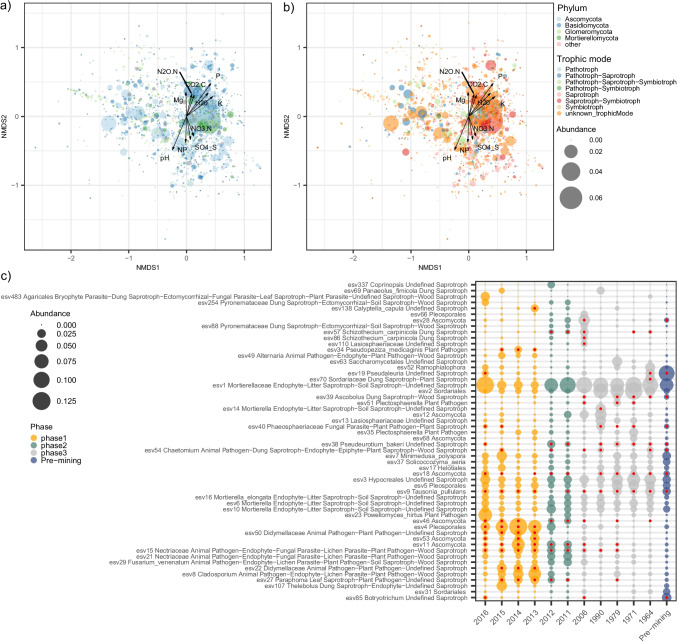
Strong turnover of the most represented fungal taxa across the recultivation chronosequence. (a, b) ESV scores in the NMDS ordination of the ESV community composition (displayed in Fig. 3a), emphasising (a) phylum and (b) trophic mode of the ESVs. Point size represents ESV relative abundance in the entire dataset. The correlation (*p* < 0.001) with soil physicochemical variables is overlaid onto the ordination. Direction and length of the arrows indicate direction of increasing value of the variable and strength of the correlation with sample scores in the ordination, respectively. (c) Aggregated ESV relative abundance per year across the chronosequence, for the combination of the ten most represented ESVs per year. A red point indicates significant association (at *p* < 0.01) between an ESV and a year when recultivation had started. The taxonomic annotation at the lowest taxonomic rank and the guild annotation is displayed

We recovered a total 2753 fungal ESVs. The NMDS ordination of Bray–Curtis community dissimilarities and variance partitioning analyses revealed that the ESV community composition successively and markedly shifted across phases, and across years of recultivation within phases (Fig. 3a, Table 1), indicating that management practices can steer the fungal community. This community turnover involved the replacement over time of the most abundant ESVs selected during each phase (Fig. 4). A few ESVs were abundant all across the chronosequence and in the pre-mining fields, but most of the ESVs were significantly more abundant in a phase than in others (Fig. 4c). The ESV community composition within fields converted to conventional agriculture (phase 3) converged towards the composition of the pre-mining community (Fig. 3a). However, according to our prediction of a stochastic reassembly of fungal taxa after mining, the ESV community composition of phase 3 fields, even of the older fields cultivated for 42 years, still differed from the pre-mining community (Fig. 3a, Table 1).

**Table 1:** PERMANOVA results of the effect of recultivation phase, year since recultivation, season and their interaction, and of the pairwise comparisons between phases of recultivation, on ESV and guild fungal community composition

	Source of variance	Df	F.Model	R2	Pr(> F)
ESV composition	Recultivation phase	3	10.8584	0.20029	**0.001**
	Year of recultivation	8	3.5125	0.17278	**0.001**
	Season	1	1.7488	0.01075	**0.024**
	Phase:Season	3	1.3655	0.02519	**0.035**
	Year:Season	7	1.1594	0.04990	0.111
Pairwise	Phase 1-Phase 2		6.839369	0.1182476	**0.001**
	Phase 1-Phase 3		15.441706	0.1601144	**0.001**
	Phase 1-Pre-mining		6.331690	0.1396747	**0.001**
	Phase 2-Phase 3		7.810195	0.1030230	**0.001**
	Phase 2-Pre-mining		6.300644	0.1950625	**0.001**
	Phase 3-Pre-mining		5.702444	0.09241844	**0.001**
Guild composition	Recultivation phase	3	17.5679	0.31681	**0.001**
	Year of recultivation	8	2.2153	0.10653	**0.003**
	Season	1	4.2558	0.02558	**0.006**
	Phase:Season	3	0.3789	0.00683	0.976
	Year:Season	7	0.6487	0.02729	0.913
Pairwise	Phase 1-Phase 2		11.68684	0.1925761	**0.0015**
	Phase 1-Phase 3		38.35827	0.3268476	**0.0015**
	Phase 1-Pre-mining		16.15684	0.3039466	**0.0015**
	Phase 2-Phase 3		7.981042	0.1050399	**0.0015**
	Phase 2-Pre-mining		7.136813	0.2153742	**0.0048**
	Phase 3-Pre-mining		1.981383	0.03417275	0.084

Numbers in bold indicate *p*-values < 0.05

One thousand eighty three ESVs (68% of the ESVs, 64% of the reads) were assigned to a fungal functional guild. Restricting the NMDS analysis to ESVs with annotation to the Funguild database revealed similar patterns as observed based on all ESVs (Fig. S1), indicating reliable interpretation of the guild community composition. The NMDS ordination of Bray–Curtis community dissimilarities and variance partitioning analyses revealed that the functional guild composition, similarly to the ESV composition, successively and markedly shifted across the phases of the recultivation and across years within phases (Fig. 3b, Table 1). However, in contrast to the ESV community composition and according to our prediction that the functional composition of the soil fungal community at the end of the chronosequence will be similar to the pre-mining state, there was no difference in guild composition between communities of the fields of phase 3 and of the pre-mining fields (Fig. 3b, Table 1).

**Fig. 5:**
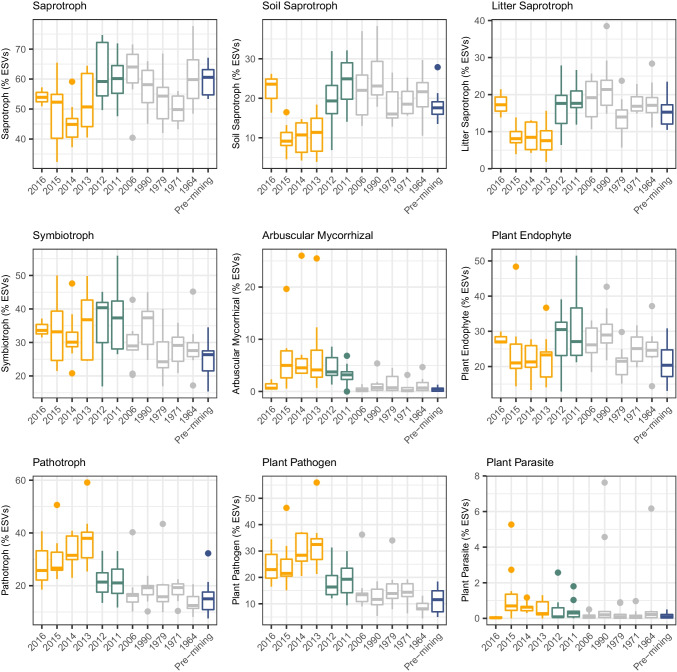
The representativity of functional guilds varies across the recultivation chronosequence. The figure depicts the median and the 25%-75% quantiles of the proportion of ESVs per sample per year. The colour of the boxes represent the phase of the recultivation chronosequence. Single dots denote outliers

A focused analysis of the functional potential of the fungal taxa revealed that AM fungi represented an increasing proportion of the fungal taxa over years after mining during phases 1 and 2 (Fig. 4a, b; Fig. 5). However, according to our second prediction, the proportion of AM fungal taxa gradually but strongly decreased after conversion to conventional agriculture (phase 3), to reach similar levels as in the pre-mining phase. Similarly, the proportion of fungal taxa with pathotrophic ability increased during the first phases of the recultivation but decreased after conversion to conventional agriculture, to reach similar levels as in the pre-mining phase (Fig. 4a, b; Fig. 5). In contrast, fungal taxa with saprotrophic ability and with endophytic ability increased in proportion, to reach a similar level as in the pre-mining state (Fig. 4a, b; Fig. 5).

The temporal change in the ESV and functional guild community composition between phases mirrored the change in soil properties, P, K and pH being the most important variables (Fig. 3, Table S1, Table S2), reflecting heavy NPK fertilisation in conventionally managed fields (phase 3 and pre-mining). The ESV and functional guild community compositions also correlated with CO_2_ potential greenhouse gas emissions. However, only the ESV composition correlated with N_2_O potential greenhouse gas emissions, consistent with its concomitant unique correlation with the NO_3_^−^ soil content.

There was only weak evidence for a change in ESV and guild composition across the two seasons that did not compare to the profound change between phases or years across the chronosequence (Table 1).

## Discussion

Soil chronosequences, in particular those in the context of agriculture, offer an interesting study case of how local-scale management can steer or not the fungal community to its pre-mining state and how decades of conventional agriculture alter the soil fungal community. Here, by characterising the soil fungal community using ITS amplicon sequencing across a 52-year chronosequence of agricultural recultivation after open-cast lignite mining of loess soil in Northern Europe, we show that functional, not taxonomic, composition reestablished the pre-mining state. We further reveal a shift in the soil fungal community as conventional agricultural practices proceed, resulting in the relative loss of plant symbionts, favouring fungi with a non-symbiotic lifestyle.

### Dynamics of the Community over time and Functional Implications

The soil fungal community rapidly responded to soil management. During excavation, loess loam of former topsoil and loess parent material are mixed, resulting in a strong dilution of soil biological properties (e.g. bacterial abundance, [23]) and nutrients of the topsoil. This was well illustrated by multiple fungal guilds contributing a very low proportion of the taxa present after soil was freshly deposited. During the first three years, AM and other plant-interacting fungi rapidly contribute to a high proportion of the taxa present. This can be explained by alfalfa, an N-fixing leguminous and AM plant, supporting the growth of AM fungi in the nutrient-poor freshly deposited soil. In addition, other plant species established in the alfalfa fields over the first three years. This probably contributed to increasing the overall fungal diversity through host plant preference of soil fungi, or host effect on the soil fungal community, driven by diverse plant–host-specific modifications of the soil properties through root exudation and litter input [38], coevolution [39] and local adaptation [40]. Overall, the dynamic of the fungal community during the first three years of recultivation suggests a rapid recovery, or resilience, of the fungal community, especially AM fungi, as observed after severe drought events in natural grasslands in fertile Chernozem soil [41].

However, conversion of alfalfa fields after the initial phase of recultivation to conventional agriculture led to a decline of plant-interacting fungi over time, AM fungi in particular, but also of pathogens and other symbiotrophs in favour of soil and litter saprotrophs and of opportunistic plant root colonising fungi (endophytes). These results are consistent with previous results at these sites focusing on AM fungi [12], supporting the utility of universal ITS primers to study AM fungi in the context of the entire fungal community and a comparison across fungal guilds [41, 42]. The replacement of AM fungi by fungi weakly interacting with plants is likely the effect of fertilisation, as previously observed in natural grasslands [9], suggesting consistent effect of fertilisation across places and ecosystems. Other factors can explain the increasing proportion of saprotrophs, such as non-AM crops (e.g. sugar beet and rapeseed) during rotations. Overall, this suggests weakened plant–fungi interactions as agricultural soils accumulate nutrients. AM fungi have been found to contribute to grain yield [43], with grain yield and nutrient content correlating positively with AM fungal richness, even in conventionally managed fields [44]. In addition, AM fungi differ among clades in access to different resources and in their potential to sustain plants facing abiotic and biotic stresses [45, 46]. Therefore, the decreased proportion of AM fungal taxa in favour of fungi weakly interacting with plants suggests a reduced potential for crops to associate with beneficial or well-adapted fungi in regard to adverse and unpredictable climatic conditions.

### Stochasticity in Taxonomic Composition and Reestablishment of the Functional Composition

The taxonomic composition of the soil fungal community after 52 years of recultivation converged with the pre-mining state but it did not reach it. It is possible that the community of the fields cultivated for more than 50 years is still a transitional community and that longer recultivation will finally result in the reestablishment of a community of similar taxonomic composition. This hypothesis is supported by differences in soil physicochemical characteristics between the fields of phase 3 and pre-mining fields. Yet, the available data point to a possible high stochasticity in the taxonomic composition reestablishment of the soil fungal community during the successional dynamic, at the highest taxonomic resolution. This result is consistent with previous results on the soil bacterial community at these sites [23], and other plant and soil communities [6].

In contrast, the functional potential of the soil fungal community of the field recultivated for the longest time period was similar to the pre-mining state. Thus, while species occurring at the end of the recultivation sequence differed from those of the pre-mining state, these species likely carried similar functional potential, in agreement with niche theory where functional traits are the basis for environmental selection [22]. This is also consistent with studies on bacteria and soil fungi showing variation in taxonomic community composition across space but strong convergence of their functions [47, 48]. Interestingly, the taxonomic and functional guild composition correlated with CO_2_ potential emissions suggesting consequences of fungal community functional shift on ecosystem properties (i.e. respiration). At the increased resolution of ESV, community composition also correlated with N_2_O potential emissions, calling for increased functional resolution to better understand the link between fungal community composition and the N cycle, and its consequences on soil functions.

Notably, we observe that decades of conventional agriculture led to a soil fungal community depleted of multiple fungal functional guilds, being dominated by soil saprotrophs, a state that is obviously easier to reach compared to a functionally diverse soil fungal community. Therefore, it remains to be assessed whether a recovery of a taxonomically and functionally diverse soil fungal community is possible with reclamation strategies in other contexts (e.g. the recovery of a natural grassland or a forest after open-mining or pollution). The increase in the ESV proportions of multiple fungal guilds during the first phase of the recultivation suggests that a recovery is possible.

### Conclusions

Both taxonomic and functional community composition showed profound shifts over time linked to soil nutrient content, reflecting the impact of heavy fertilisation. The rapidly increasing contribution of AM and other plant-interacting fungi rapidly to the richness of the soil fungal community during the first three years of alfalfa recultivation suggests a recovery, or resilience, of the fungal community, especially AM fungi, in the fertile loess soil, which probably contributed to the speed of the recovery. However, conversion to conventional agriculture led to a decline of AM fungi replaced by soil saprotrophs, suggesting a decreased potential for crops to rely on these important plant symbionts for nutrition and to face unpredictable adverse climatic conditions. Taxonomic composition analysed at the highest taxonomic resolution did not reach the pre-mining state even after more than 50 years of recultivation, but functional composition did. Thus, local-scale management, under conditions comparable to those in this study, can steer the fungal community to its functional pre-mining state. But it remains to be assessed whether a recovery of a taxonomically and functionally diverse soil fungal community is possible with reclamation strategies in other contexts.

## Supplementary Information

Below is the link to the electronic supplementary material.

Supplementary file1 (ODT 85.2 KB)

## Data Availability

Raw sequencing reads are available at ENA under study project PRJEB51095. Fungal ESV sequences are available at ENA under accession numbers OV986018-OV989728. The ESV contingency table, the sample metadata and the taxonomic and functional annotation of ESVs, are available as .RDS R files at figshare (10.6084/m9.figshare.20160578).
