# Supplementary material for: Functional, not Taxonomic, Composition of Soil Fungi Reestablishes to Pre-mining Initial State After 52 Years of Recultivation
Source: Microb Ecol. 2022 Jul 12;86(1):213–23. doi: 10.1007/s00248-022-02058-w (PMC10293406; doi:10.1007/s00248-022-02058-w)
Supplement: Supplementary file 1 [file 248_2022_2058_MOESM1_ESM.odt]

Supplementary Material for «Functional, not taxonomic, composition of soil fungi reestablishes to pre-mining initial state after 52 years of recultivation»

Julien Roy 1,2,∗ , Rüdiger Reichel 3 , Nicolas Brüggemann 3 , and Matthias C Rillig 1,2

1 Institut für Biologie, Ökologie der Pflanzen, Freie Universität Berlin, 14195 Berlin, Germany,

2 Berlin-Brandenburg Institute of Advanced Biodiversity Research, 14195 Berlin, Germany

3 Forschungszentrum Jülich GmbH, Institute of Bio- and Geosciences, Agrosphere (IBG-3), 52425 Jülich, Germany

* Author for correspondance : royjulien@zedat.fu-berlin.de

Figure S1. Similar patterns in the change in the taxonomic community composition of soil fungi across the recultivation chronosequence restricting the analysis to ESVs with guild annotation in Funguild. The figure depicts the NMDS ordination of ESV community composition of soil fungi across the reclamation chronosequence, to be compared to Figure 3, panel a. Significant correlations between NMDS scores and soil parameters (p <0.001) are displayed.

Table S1. Correlation between soil physicochemical variables and community scores in the NMDS ordination of OTU and guild community composition. Signif. codes:  0 ‘***’ 0.001 ‘**’ 0.01 ‘*’ 0.05 ‘.’ 0.1 ‘ ’ 1.

|  |  | NMDS1 | NMDS2 | r2 | Pr(>r) |
| --- | --- | --- | --- | --- | --- |
| OTU composition | gravimetricH2O | -0.39615 | -0.91819 | 0.1153 | 0.001 *** |
|  | pH_CaCl2 | 0.44809 | 0.89399 | 0.2980 | 0.001 *** |
|  | NH4.N | 0.13262 | 0.99117 | 0.0230 | 0.260 |
|  | NO3.N | -0.40257 | 0.91539 | 0.0992 | 0.003  ** |
|  | P_CAL | -0.66146 | -0.74998 | 0.4120 | 0.001 *** |
|  | K_CAL | -0.84547 | -0.53402 | 0.2946 | 0.001 *** |
|  | Mg_CaCl2 | 0.03059 | -0.99953 | 0.1253 | 0.001 *** |
|  | SO4_S | -0.20830 | 0.97807 | 0.1181 | 0.001 *** |
|  | CO2.C | -0.74345 | -0.66879 | 0.2603 | 0.001 *** |
|  | N2O.N | -0.27158 | -0.96241 | 0.1131 | 0.002 ** |
|  | NP | 0.03944 | 0.99922 | 0.1415 | 0.001 *** |
| Guild composition | gravimetricH2O | 0.95845 | -0.28527 | 0.0578 | 0.042 * |
|  | pH_CaCl2 | -0.89822 | -0.43954 | 0.1808 | 0.001 *** |
|  | NH4.N | -0.76238 | -0.64712 | 0.0138 | 0.473 |
|  | NO3.N | 0.74059 | -0.67196 | 0.0153 | 0.433 |
|  | P_CAL | 0.99992 | 0.01239 | 0.3136 | 0.001 *** |
|  | K_CAL | 0.94817 | -0.31777 | 0.2227 | 0.001 *** |
|  | Mg_CaCl2 | 0.46482 | 0.88540 | 0.0250 | 0.232 |
|  | SO4_S | 0.53330 | -0.84593 | 0.0100 | 0.592 |
|  | CO2.C | 0.95023 | -0.31154 | 0.2490 | 0.001 *** |
|  | N2O.N | 0.99911 | 0.04214 | 0.0533 | 0.053 . |
|  | NP | -0.84697 | -0.53164 | 0.0174 | 0.374 |

Table S2. Unique part of variance in community composition explained by each soil physicochemical variable. Signif. codes:  0 ‘***’ 0.001 ‘**’ 0.01 ‘*’ 0.05 ‘.’ 0.1 ‘ ’ 1.

|  |  | Df | SumOfSqs | R2 | F | Pr(>F) |
| --- | --- | --- | --- | --- | --- | --- |
| OTU composition | gravimetricH2O | 1 | 0.2656 | 0.00914 | 1.1964 | 0.203 |
|  | pH_CaCl2 | 1 | 0.8282 | 0.02849 | 3.7305 | 0.001 *** |
|  | NH4.N | 1 | 0.4193 | 0.01442 | 1.8886 | 0.011 * |
|  | NO3.N | 1 | 0.3389 | 0.01166 | 1.5265 | 0.071 . |
|  | P_CAL | 1 | 0.5052 | 0.01738 | 2.2757 | 0.006 ** |
|  | K_CAL | 1 | 0.5084 | 0.01749 | 2.2900 | 0.004 ** |
|  | Mg_CaCl2 | 1 | 0.6459 | 0.02222 | 2.9096 | 0.001 *** |
|  | SO4_S | 1 | 0.3801 | 0.01307 | 1.7122 | 0.040 * |
|  | NP | 1 | 0.3817 | 0.01313 | 1.7195 | 0.034 * |
|  | Residuals | 101 | 22.4219 | 0.77126 |  |  |
|  | Total | 110 | 29.0717 | 1.00000 |  |  |
| Guild composition | gravimetricH2O | 1 | 0.01338 | 0.00481 | 0.6664 | 0.591 |
|  | pH_CaCl2 | 1 | 0.11947 | 0.04292 | 5.9515 | 0.001 *** |
|  | NH4.N | 1 | 0.06426 | 0.02309 | 3.2011 | 0.025 * |
|  | NO3.N | 1 | 0.02812 | 0.01010 | 1.4008 | 0.209 |
|  | P_CAL | 1 | 0.07269 | 0.02611 | 3.6209 | 0.018 * |
|  | K_CAL | 1 | 0.02117 | 0.00760 | 1.0544 | 0.345 |
|  | Mg_CaCl2 | 1 | 0.08257 | 0.02966 | 4.1129 | 0.007 ** |
|  | SO4_S | 1 | 0.03576 | 0.01285 | 1.7811 | 0.119 |
|  | NP | 1 | 0.01221 | 0.00439 | 0.6084 | 0.662 |
|  | Residuals | 99 | 1.98737 | 0.71397 |  |  |
|  | Total | 108 | 2.78357 | 1.00000 |  |  |
